# MicroRNA profiles in serum samples from Acute-On-Chronic Liver Failure patients and miR-25-3p as a potential biomarker for survival prediction

**DOI:** 10.1038/s41598-019-56630-5

**Published:** 2020-01-09

**Authors:** Júlia Cisilotto, Alex Evangelista do Amaral, Daiane Rosolen, Michele Patrícia Rode, Adny Henrique Silva, Evelyn Winter, Telma Erotides da Silva, Josiane Fischer, Camila Matiollo, Elayne Cristina de Morais Rateke, Janaína Luz Narciso-Schiavon, Leonardo de Lucca Schiavon, Tânia Beatriz Creczynski-Pasa

**Affiliations:** 1 Department of Pharmaceutical Sciences, Federal University of Santa Catarina, Florianopolis, 88040-900 SC Brazil; 2 Department of Biochemistry, Federal University of Santa Catarina, Florianopolis, 88040-900 SC Brazil; 3 Department of Agriculture, Biodiversity and Forestry, Federal University of Santa Catarina, Curitibanos, 89520-000 SC Brazil; 4 Department of Internal Medicine, Division of Gastroenterology, Federal University of Santa Catarina, Florianópolis, 88040-900 SC Brazil

**Keywords:** Molecular biology, Prognostic markers, Hepatology

## Abstract

Acute-on-chronic liver failure (ACLF) is a condition characterized by acute decompensation of cirrhosis, associated with organ failure(s), and high short-term mortality. The microRNAs or miRNAs are small non-coding RNA molecules, stable in circulating samples such as biological fluids, and the difference in expression levels may indicate the presence, absence and/or stage of the disease. We analyzed here the miRNA profiling to identify potential diagnostic or prognostic biomarkers for ACLF. The major miRNAs discovered were validated in a cohort of patients with acute decompensation of cirrhosis grouped in no ACLF or ACLF according to EASL-CLIF definition. Relationship between serum miRNAs and variables associated with liver-damage and survival outcomes were verified to identify possible prognostic markers. Our results showed twenty altered miRNAs between no ACLF and ACLF patients, and twenty-seven in patients who died in 30 days compared with who survived. In validation phase, miR-223-3p and miR-25-3p were significantly altered in ACLF patients and in those who died in 30 days. miR-223-3p and miR-25-3p expression were associated with the lowest survival in 30 days. The decrease in miR-223-3p and miR-25-3p expression was associated with the presence of ACLF and poor prognosis. Of these, miR-25-3p was independently related to ACLF and 30-day mortality.

## Introduction

The natural history of cirrhosis is usually characterized by a long-standing compensated phase followed by a transition to the decompensated disease, identified by the occurrence of specific complications of cirrhosis, such as ascites, variceal bleeding, and hepatic encephalopathy^1^. Patients with both compensated or decompensated cirrhosis are at risk of progression to acute-on-chronic liver failure (ACLF), a condition characterized by an acute deterioration of the liver function and characterized by progression to extrahepatic organ failure and high short-term mortality^2,3^. Although a precise definition is still lacking, the European Association for the Study of the Liver-Chronic Liver Failure (EASL-CLIF) Consortium definition is one of the most validated criteria for ACLF in patients with cirrhosis and it is based on a modified version of SOFA score called CLIF-SOFA^2,4^. According to a recently published study^5^, mortality rates in ACLF patients are 25.5% and 40% in 28 and 90 days of admission, respectively. Therefore, searching for new biomarkers associated with the presence of ACLF and prognosis of patients with acute decompensation of cirrhosis may improve clinical decision and help to implement risk-adapted treatment strategies. In recent years, miRNAs have been studied as promising biomarkers for the diagnosis and prognostic in many clinical scenarios^6–8^, including liver diseases^9^. The miRNAs are a group of small non-coding RNAs, with approximately 22 nucleotides, which post-transcriptionally regulate gene expression usually by binding to the 3′ UTR of the mRNA^10,11^. The miRNAs have already been detected in different biological fluids such as plasma, serum, tears, breast milk, cerebrospinal fluid, saliva, urine, and others^12,13^. It has been shown that miRNAs are stable in serum/plasma and can be directly detected in these fluids, which improve their application as biomarkers. In addition, miRNAs are resistant to RNaseA digestion and other harsh conditions, which potentially support its usage in clinical test^14^.

Although there are data regarding different miRNAs in patients with hepatitis B virus (HBV)-related ACLF^15,16^, to our knowledge, no study investigated miRNAs in patients with acute decompensation of cirrhosis or ACLF. Thus, in the present study, we aimed to evaluate the miRNAs expression in serum samples from patients with acute decompensation of cirrhosis, identifying possible biomarkers of ACLF and prognosis.

## Results

### miRNA selection from the microarray panel

At the first phase, 35 serum samples were evaluated in miRNAs microarray analysis. The mean age of these patients was 53.6 ± 8.8 years, with male predominance (74.3%). The most common etiologic factor of cirrhosis was alcohol consumption (62.9%) followed by hepatitis C (40.0%). Upon admission, ascites was observed in 60.0% of cases and bacterial infection in 45.7%. The most frequent medications regularly used by the included subjects were propranolol (42.9%), spironolactone (42.9%), furosemide (34.3%), lactulose (28.6%) and omeprazole (40.0%). Among the 35 patients, 20 fulfilled the criteria for ACLF and 7 died within 30 days (Table 1).

**Table 1:** Characteristics of the included patients in miRNA microarray analysis.

Variables		Patients (%)	Values Mean ± SD
**Age, years**		35 (100.0)	53.6 ± 8.8
**Gender**	Male	26 (74.3)	
	Female	9 (25.7)	
**Etiology of cirrhosis**			
Hepatitis C	Male	10 (28.6)	
	Female	4 (11.4)	
Hepatitis B	Male	2 (5.7)	
	Female	1 (2.9)	
Alcohol	Male	21 (60.0)	
	Female	1 (2.9)	
**ACLF**		20 (57.1)	
**Bacterial infection (first 48 h)**		16 (45.7)	
**Ascites at admission**		21 (60.0)	
**Medications at admission**			
Propranolol		15 (42.9)	
Spironolactone		15 (42.9)	
Furosemide		12 (34.3)	
Lactulose		10 (28.6)	
Omeprazole		14 (40.0)	
Oral hypoglycemic*		4 (11.4)	
Insulin		3 (8.6)	
**Death in 30 days**		7 (20.0)	
**Biochemical data**			
Total bilirubin (mg/dL)		35 (100.0)	4.9 ± 7.9
INR		33 (94.3)	1.5 ± 0.3
CRP (mg/L)		28 (80.0)	35.0 ± 45.8
Creatinine (mg/dL)		26 (74.3)	1.5 ± 0.8
Albumin (g/dL)		34 (97.1)	2.4 ± 0.5
Sodium (mEq/L)		33 (94.3)	134.4 ± 4.9
Total leukocyte (mm^3^)		32 (91.4)	7163.8 ± 5923.0

ACLF. acute-on-chronic liver failure; SD. standard deviation; INR. international normalized ratio; CRP. C-reactive protein. *Metformin, glibenclamide and glimepiride.

As showed in Table 2, miRNA expression from microarray analysis was evaluated according to the presence of ACLF and 30-day mortality. The expression of 20 miRNAs was decreased by at least 6-fold in ACLF patients compared to no ACLF (fold-change (FC) ≥ 6). Among them, expression of the miRNAs let-7a-5p, let-7g-5p, miR-106b-5p, miR-107, miR-126-3p, miR-17-5p, miR-20a-5p, miR-25-3p and miR-451a was decreased by at least 10-fold in ACLF patients (FC ≥ 10). When miRNA expression was evaluated according to survival, expression of 27 miRNAs was reduced in those who died within 30 days (FC ≥ 6). Expression of miRNAs let-7a-5p, let-7g-5p, miR-106b-5p, miR-126-3p, miR-15b-5p, miR-17-5p, miR-199a-3p, miR-20a-5p, miR-223-3p, miR-25-3p and miR-575 were decreased by at least 10-fold in individuals who died in relation to survivors (FC ≥ 10).

**Table 2:** miRNA expression from microarray analysis according to the presence of ACLF and 30-day mortality.

no ACLF *vs* ACLF patients				Survival *vs* Death in 30 days			
miRNA	P-value	FC	Regulation	miRNA	P-value	FC	Regulation
let-7a-5p	0.004	10	down	let-7a-5p	0.009	13	down
let-7b-5p	0.007	6	down	let-7f-5p	0.012	9	down
let-7g-5p	0.009	14	down	let-7g-5p	0.008	18	down
miR-106b-5p	0.007	10	down	miR-106b-5p	0.006	13	down
miR-107	0.001	11	down	miR-126-3p	0.004	14	down
miR-126-3p	0.006	14	down	miR-146a-5p	0.012	9	down
miR-142-3p	0.009	8	down	miR-150-5p	0.015	8	down
miR-150-5p	0.005	7	down	miR-15b-5p	0.017	10	down
miR-15b-5p	0.002	9	down	miR-16-5p	0.026	8	down
miR-16-5p	0.006	8	down	miR-17-5p	0.011	12	down
miR-17-5p	0.004	15	down	miR-199a-3p	0.009	10	down
miR-20a-5p	0.012	18	down	miR-19a-3p	0.013	8	down
miR-223-3p	0.002	7	down	miR-19b-3p	0.021	7	down
miR-25-3p	0.005	16	down	miR-20a-5p	0.043	15	down
miR-27a-3p	0.008	7	down	miR-223-3p	0.003	10	down
miR-328-5p	0.003	6	down	miR-23a-3p	0.011	8	down
miR-451a	0.004	10	down	miR-24-3p	0.092	9	down
miR-4530	0.002	7	down	miR-25-3p	0.026	11	down
miR-5703	0.001	7	down	miR-328-5p	0.017	6	down
miR-630	0.001	7	down	miR-4515	0.018	8	down
				miR-451a	0.029	8	down
				miR-4530	0.009	7	down
				miR-4741	0.004	7	down
				miR-575	0.010	10	down
				miR-5787	0.019	7	down
				miR-6090	0.019	7	down
				miR-6752-5p	0.008	6	down

It was considered only miRNAs with fold-change (FC) ≥ 6 and P-value < 0.05.

**Figure 1:**
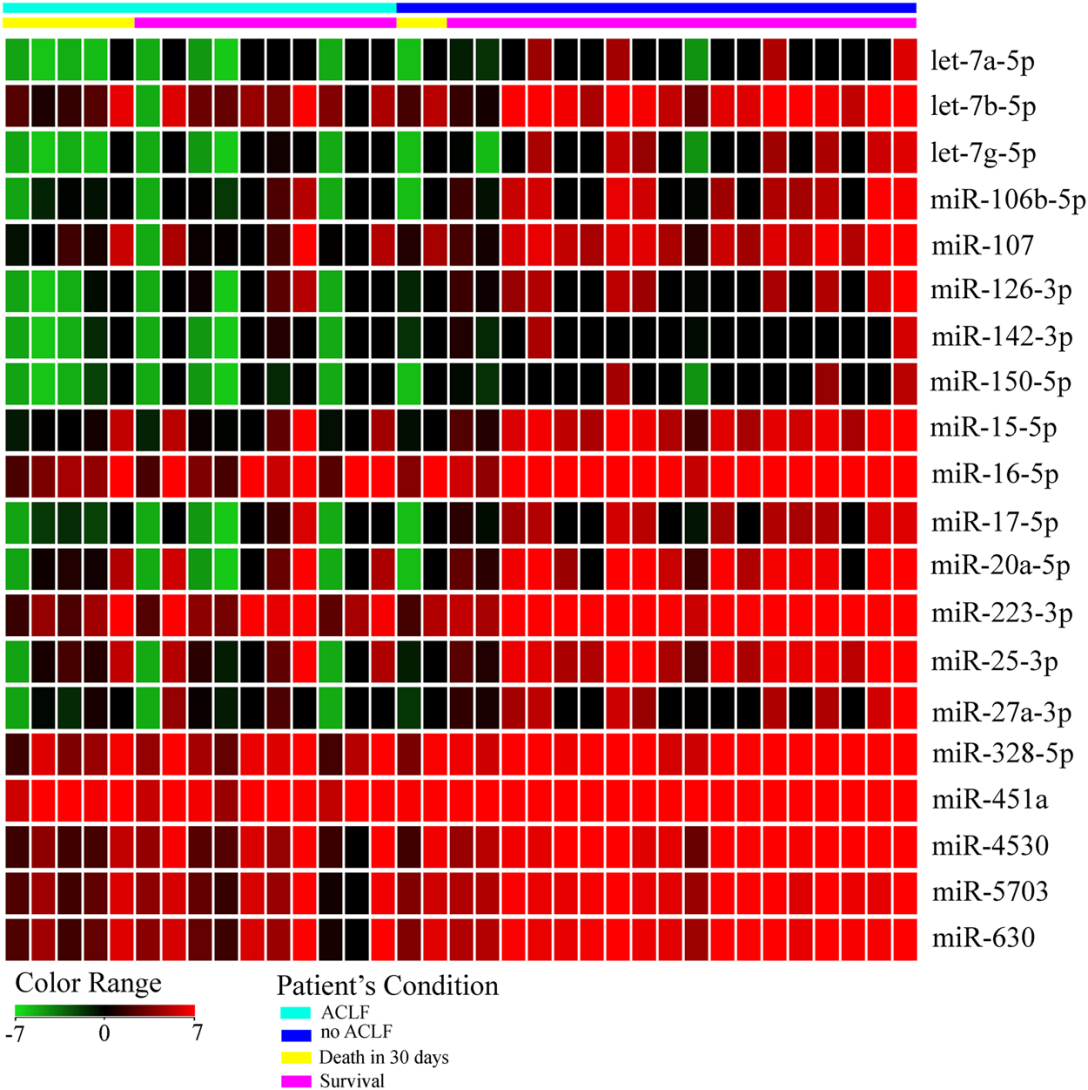
Heat map of differentially expressed miRNAs according to the presence of ACLF and survival. Heat map colors correspond to the level of miRNA expression as indicated in the color range.

When data of survivors’ patients and those who died within 30 days were compared using a FC less than 6 it was possible to detect the following upregulated miRNAs: miR-1180-3p (FC = 3; P = 0.02); miR-1228-5p (FC = 3; P = 0.02); miR-670-5p (FC = 3; P = 0.04); miR-6847-3p (FC = 3; P = 0.02); miR-7844-5p (FC = 3; P = 0.04). Figure 1 shows the expression intensity of deregulated miRNAs (FC ≥ 6 and P < 0.05) in samples from patients with no ACLF, ACLF, and those who died. ACLF patients exhibited a reduction in expression intensity mainly in the miRNAs let-7a-5p, let-7g-5p, miR-106b-5p, miR-107, miR-126-3p, miR-142-3p, miR-150-5p, miR-17-5p, miR-20a-5p, miR-25-3p and miR-27a-3p. It was also checked whether the most frequent medications regularly used by the patients (propranolol, spironolactone, furosemide, lactulose, norfloxacin, omeprazole, oral hypoglycemic and insulin) would alter miRNA expression. As can be seen in Supplementary Fig. S1, no medication-related changes in miRNAs expression (P < 0.05 and FC > 2) were observed.

Based on the magnitude of expression difference between patients with and without ACLF in the microarray analysis and in literature reports^17–23^ miRNAs miR-106b-5p, miR-126-3p, miR-20a-5p, miR-223-3p, and miR-25-3p were selected to be further validated by RT-qPCR. Some miRNAs that had high FC values such as let-7a-5p and let-7g-5p were not detected in most microarray samples and were therefore not included in the validation step.

### Characteristics of included patients and associations between miRNA expression and variables of interest

One hundred and thirty-nine subjects with acute decompensation of cirrhosis were included in the validation step for RT-qPCR. Table 3 shows the main characteristics of patients. The mean age was 55.3 ± 11.2 years and a male predominance was observed (70.5%). The most common etiology of cirrhosis was alcohol abuse (50.4%) followed by hepatitis C (37.4%), and hepatitis B (7.2%). The main medications regularly used by patients prior to the blood sampling were propranolol (36.7%), spironolactone (31.7%), furosemide (21.6%), lactulose (20.9%), norfloxacin (11.5%) and omeprazole (35.5%). The mean MELD score was 17.5 ± 6.9 and 35 patients (25.1%) fulfilled ACLF criteria.

**Table 3:** Characteristics of included patients and factors associated with ACLF at enrollment.

	All (n = 139)	no ACLF (n = 104)	ACLF (n = 35)	P-value
**Age (years), mean ± SD**	55.3 ± 11.2	55.4 ± 11.8	54.8 ± 9.59	0.783
**Male Gender, n (%)**	98 (70.5)	73 (70.2)	25 (71.4)	0.890
**Etiology of cirrhosis**, **n (%)**^♦^				
Hepatitis C	52 (37.4)	34 (32.7)	18 (51.4)	0.048*
Hepatitis B	10 (7.2)	7 (6.7)	3 (8.6)	0.715
Alcohol	70 (50.4)	56 (53.8)	14 (40.0)	0.156
**Complication at admission**, **n (%)**				
Ascites	69 (49.6)	40 (38.5)	29 (82.9)	<0.001***
Hepatic encephalopathy	63 (45.3)	37 (35.6)	26 (74.3)	<0.001***
Gastrointestinal bleeding	48 (34.5)	43 (41.3)	5 (14.3)	0.004**
Bacterial infection	69 (49.6)	48 (46.2)	21 (60)	0.156
**Medications at admission**, **n (%)**				
Propranolol	51 (36.7)	42 (40.4)	9 (25.7)	0.119
Spironolactone	44 (31.7)	30 (28.8)	14 (40)	0.220
Furosemide	30 (21.6)	17 (16.3)	13 (37.1)	0.010*
Lactulose	29 (20.9)	18 (17.3)	11 (31.4)	0.075
Norfloxacin	16 (11.5)	8 (7.7)	8 (22.9)	0.015*
Omeprazole	49 (35.5)	39 (37.9)	10 (28.6)	0.321
Oral hypoglycemic^••^	19 (13.8)	17 (16.5)	2 (5.7)	0.109
Insulin	15 (11.0)	10 (9.9)	5 (14.3)	0.475
**Child-Pugh Classification**, **n (%)**				
Child-Pugh A	11 (10.6)	11 (8.0)	0 (0.0)	0.065
Child-Pugh B	71 (51.4)	60 (57.7)	11 (32.4)	0.017*
Child-Pugh C	56 (40.6)	33 (31.7)	23 (67.6)	<0.001***
**MELD score, mean ± SD**	17.5 ± 6.9	14.8 ± 4.4	25.4 ± 7.2	<0.001***
**Biochemical data**				
Creatinine (mg/dL), median	1.1	1.0	2.4	<0.001
Total leukocyte (mm^3^), median	6700.0	6540.0	8450.0	0.072
Total bilirubin (mg/dL), median	2.1	2.0	3.5	0.029*
Albumin (g/dL), mean ± SD	2.4 ± 0.6	2.4 ± 0.6	2.4 ± 0.6	0.893
INR, median	1.5	1.4	1.6	0.034*
Sodium (mEq/L), mean ± SD	134.9 ± 5.5	136.2 ± 5.0	131.5 ± 5.5	<0.001***
CRP (mg/L), median	19.6	18.0	26.4	0.029*
**MAP (mmHg), mean ± SD**	83.4 ± 16.9	85.4 ± 15.5	77.5 ± 20.0	0.020*

ACLF. acute-on-chronic liver failure; SD. standard deviation; INR. international normalized ratio; CRP. C-reactive protein; MELD. model for end-stage liver disease; MAP. Mean Arterial Pressure. ^♦^Of the 52 patients with hepatitis C as etiology of cirrhosis 39 (75%) were male and 13 (25%) female; Of the 10 patients with hepatitis B as etiology of cirrhosis 8 (80%) were male and 2 (20%) female; Of the 70 patients with alcohol use as etiology of cirrhosis 65 (92.9%) were male and 5 (7.1%) female. ^••^Metformin, glibenclamide and glimepiride. *P < 0.05; **P < 0.01 and ***P < 0.001.

**Figure 2:**
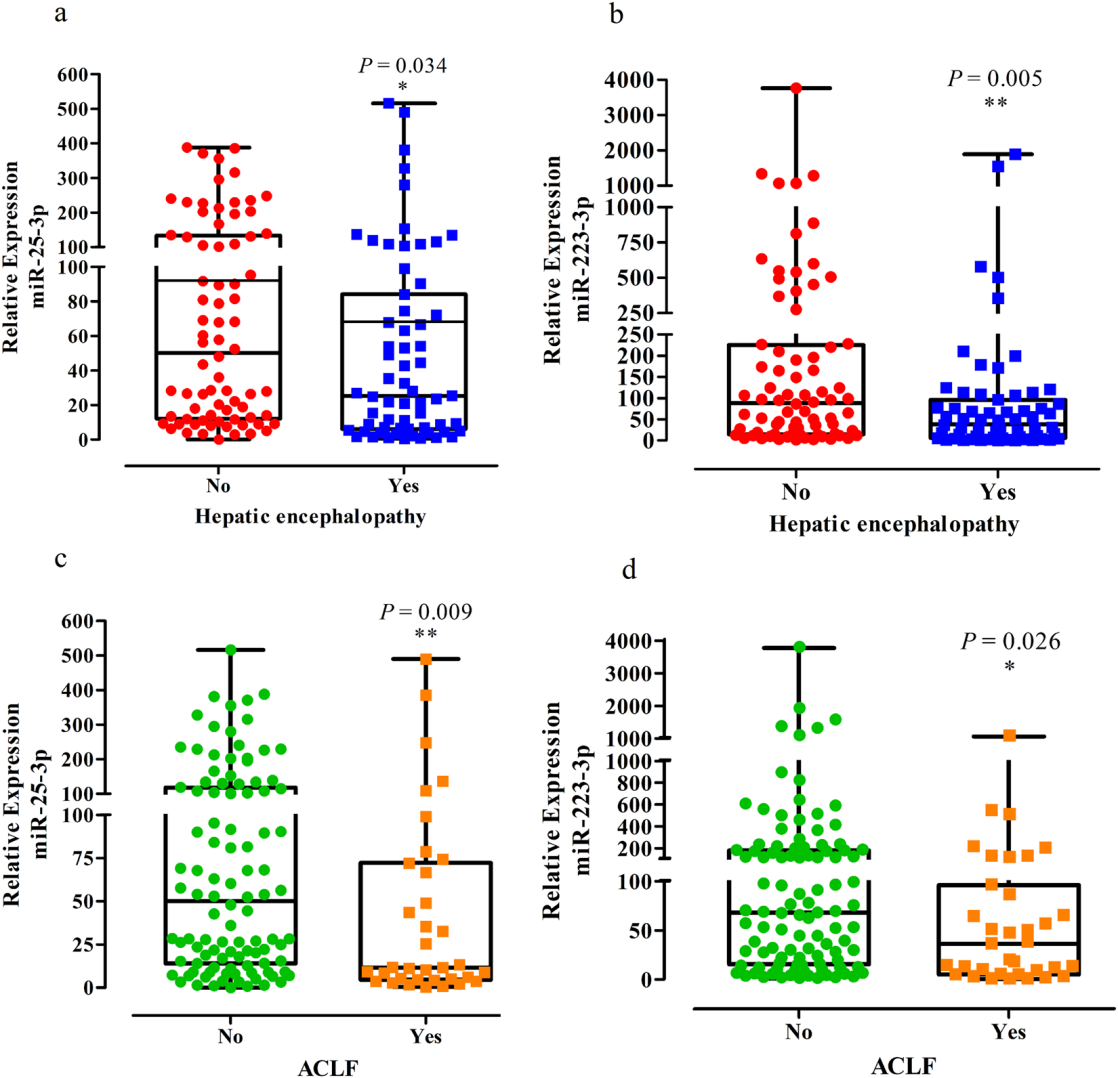
Relative expression of miR-25-3p and miR-223-3p in patients with hepatic encephalopathy (**a,b**) and ACLF patients (**c,d**). In the graphs, the horizontal lines of Box plot represent the median per group, minimum and maximum values. Statistical differences among the groups were calculated using Mann-Whitney U tests. Asterisks indicate whether there were significant differences among the groups. *P < 0.05; **P < 0.01.

When miR-106b-5p, miR-126-3p, miR-20a-5p, miR-223-3p and miR-25-3p were evaluated according to the study variables, the expression of miR-223-3p and miR-25-5p was significantly decreased in patients with hepatic encephalopathy at admission (P = 0.005 and 0.034, respectively) and in ACLF individuals (P = 0.026 and 0.009, respectively; Supplementary Table S1 and Fig. 2). Moreover, when evaluating only ACLF patients (n = 35), no significant differences were observed in the expressions of miR-25-3p (median:10.3 *vs* 13.4, respectively; P = 0.250) and miR-223-3p (median: 28.1 *vs* 64.8, respectively; P = 0.117) among those with (n = 26, 74.3%) and without hepatic encephalopathy. Similarly, when only no ACLF patients (n = 104) were considered, no differences were observed for miR-25-3p (median: 42.7 *vs* 56.3, respectively; P = 0.352) and miR-223-3p (median: 56.7 *vs* 90.6, respectively; P = 0.092) among those with (n = 37, 35.6%) and without hepatic encephalopathy. Regarding correlation analysis, miR-106b-5p negatively correlated with total leukocyte count (r = −0.212, P = 0.012) and miR-20a-5p negatively correlated with serum creatinine (r = −0.212, P = 0.012). miR-223-3p expression was negatively correlated with age (r = −0.193, P = 0.025), creatinine levels (r = −0.302, P < 0.001), and CLIF-SOFA (r = −0.186, P = 0.034). Likewise, miR-25-3p expression was negatively correlated with serum creatinine (r = −0.341, P < 0.001), CLIF-SOFA (r = −0.196, P = 0.026), and MELD score (r = −0.185, P = 0.029; Supplementary Table S2).

### Factors associated with ACLF

As shown in Table 3, ACLF patients showed a higher prevalence of hepatic encephalopathy (74.3% *vs*. 35.6%, P < 0.001) and ascites (82.9% *vs*. 38.5%, P < 0.001), and lower prevalence of gastrointestinal bleeding (14.3% *vs*. 41.3%, P = 0.004). Regarding biochemical parameters, ACLF patients exhibited higher median creatinine (P < 0.001), total bilirubin (P = 0.029), INR (P = 0.034), CRP (P = 0.029), and lower mean sodium levels (P < 0.001). ACLF was also related to lower mean arterial pressure (P = 0.020) and higher MELD score (P < 0.001). As described before, subjects with ACLF at admission exhibited decreased expression of miR-223-3p and miR-25-5p (Table S1).

A logistic regression analysis was performed to investigate factors independently associated with ACLF including the following covariates with P < 0.05 in the bivariate analysis: sodium levels, hepatitis C, Child-Pugh C, miR-223-3p and miR-25-3p expression. In this analysis, ACLF at admission was independently associated with lower sodium levels (OR = 0.82, 95% CI: 0.74–0.91, P < 0.001), Child-Pugh C (OR = 3.48, 95% CI: 1.34–9.01, P = 0.010), hepatitis C (OR = 3.69, 95% CI: 1.35–10.04, P = 0.011) and miR-25-3p expression (OR = 0.99, 95% CI: 0.98–0.99, P = 0.040).

### miRNAs as prognostic markers

During the first 30 days of follow-up, 31 patients died (22%). Survival analysis was performed to identify the association between studied miRNAs and 30-day mortality. Expressions of miR-223-3p (HR = 0.99, 95% CI: 0.98–0.99, P = 0.030) and miR-25-3p (HR = 0.99, 95% CI: 0.98–0.99, P = 0.013) were associated with 30-day mortality in the univariate Cox regression analysis. As expected, Child-Pugh C (HR = 3.99, 95% CI: 1.83–8.72, P = 0.001) and ACLF (HR = 4.78, 95% CI: 2.35–9.73, P < 0.001) were also related to shorter survival. Expressions of miR-106b-5p, miR-126-3p and miR-20a-5p were not associated with prognosis (Table 4). A multiple Cox regression analysis was conducted including the expression of miR-223-3p and miR-25-3p, along with Child-Pugh C and ACLF. As shown in Table 4, decreased miR-25-3p expression status (HR = 0.99, 95% CI: 0.98–0.99, P = 0.044), Child-Pugh C (HR = 2.92, 95% CI: 1.28–6.66, P = 0.011), and ACLF (HR = 2.69, 95% CI: 1.25–5.80, P = 0.012) were independently predictors of 30-day mortality.

**Table 4:** Univariate and Multivariate Cox regression analysis of variables associated with 30-day survival among hospitalized patients with acute decompensation of cirrhosis.

Variables	Univariate analysis			Multivariate analysis		
	HR	95% CI	P-value	HR	95% CI	P-value
miR-106b-5p	0.98	0.95 – 1.00	0.051	—	—	—
miR-126-3p	0.98	0.95 – 1.02	0.279	—	—	—
miR-20a-5p	0.99	0.99 – 1.00	0.052	—	—	—
miR-223-3p	0.99	0.98 – 0.99	0.030	—	—	—
miR-25-3p	0.99	0.98 – 0.99	0.013	0.99	0.98 – 0.99	0.044
Child-Pugh C	3.99	1.83 – 8.72	0.001	2.92	1.28 – 6.66	0.011
ACLF	4.78	2.35 – 9.73	<0.001	2.69	1.25 – 5.80	0.012

CI. confidence interval; HR. hazard ratio.

**Figure 3:**
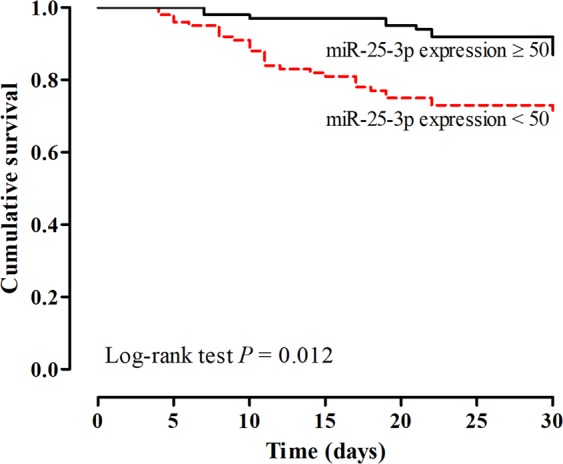
Cumulative 30-day survival of patients with cirrhosis according to miR-25-3p expression categorized in 50. The Kaplan-Meier survival probability at 30 days was 87% for patients with miR-25-3p expression ≥ 50 and 70% for those with miR-25-3p expression < 50 (red dotted line) (P = 0.012).

Figure 3 shows the Kaplan-Meier curves for mortality during the follow-up period, according to miR-25-3p expression categorized in 50. The Kaplan-Meier survival probability at 30 days was 87% for patients with miR-25-3p expression ≥ 50 and 70% for those with miR-25-3p expression < 50 (red dotted line) (P = 0.012).

### Bioinformatic analysis of the miR-25-3p

The results showed 1873 targets predicted for the miR-25-3p. Among the target genes, some are related with inflammation, such as, intercellular adhesion molecule 1 (ICAM-1 or CADM1); C-X-C motif chemokine 5 (CXCL5); TNF receptor-associated factor 3 (TRAF3); interleukin 8 (IL8); interleukin 6 signal transducer (IL6ST); interleukin 17 receptor D (IL17RD) and interleukin 15 (IL15). The Supplementary Fig. S2 shows some significant pathways (P < 0.05) within the list of miR-25-3p related genes. Among 1873 genes, 32.3% are associated with the vascular endothelial growth factor (VEGF) and the VEGF receptor (VEGFR) signaling network; 32.2% and 32.3% with interleukin 5 (IL5) and 3 (IL3) signaling events, respectively; 32.3% with interferon gamma (INF-γ); 8.2% with tumor necrosis factor (TNF) receptors and 33.8% with TNF-related apoptosis-inducing ligand (TRAIL) signaling pathway.

## Discussion

Over the last decade, significant advances were achieved in definition and understanding of the pathogenesis of ACLF. However, data about the role of miRNAs in this context is still lacking. In this study, we sought to identify miRNAs related to the presence of ACLF and prognosis in patients hospitalized for acute decompensation of cirrhosis. The first phase of the study aimed at evaluating the miRNAs panel differently expressed in ACLF patients by using a microarray-based approach, which allows the simultaneous evaluation of the countless miRNAs expression. In this analysis, it was possible to identify 20 altered miRNAs in ACLF patients and 27 miRNAs related to 30-day mortality. Among these, 5 miRNAs (miR-106b-5p; miR-126-3p; miR-20a-5p; miR-223-3p; miR-25-3p) were selected for RT-qPCR validation. These miRNAs were chosen for validation based on (i) the results obtained in microarray analysis (P-value and fold-change); (ii) the number of samples in which a specific miRNA was detected; (iii) and previously published data regarding the miRNAs profile in decompensated cirrhosis^17,18^, inflammation^19,20^, and sepsis^21–23^.

When miRNAs serum expressions assessed by RT-qPCR were evaluated according to study variables, no relationship was observed between miR-126-3p and any of the analyzed parameters. There are no previous data regarding circulating miR-126-3p and liver cirrhosis. This miRNA was previously shown to be downregulated in non-cirrhotic patients with sepsis and was negatively correlated with inflammation biomarkers, blood urea nitrogen levels, and total bilirubin^23^. It is possible that microarray results regarding miR-126-3p reflect the inflammatory component of ACLF, even though we were not able to demonstrate any relationship between this miRNA and parameters such as leukocyte count and CRP. In the present study, we also observed no significant correlations between miR-106b-5p and most of the studied variables, except the leukocyte count. Contrary to our results, Jin *et al*.^18^ showed that miR-106b-5p was downregulated in plasma from patients with HBV-related cirrhosis classified as Child-Pugh B/C (n = 47) compared to Child-Pugh A patients (n = 53). This discrepancy may be, at least in part, related to the impact of the etiological factor of liver disease in circulating miRNAs. The Chinese study only included HBV-infected patients, whereas, in our cohort, this etiological factor was present in the minority of the patients. Circulating miR-20a-5p was also not related to most of the study variables, but a negative correlation was observed between this miRNA and creatinine levels. miR-20a-5p was also not previously studied in patients with liver cirrhosis. However, it was shown to be downregulated in chronic kidney disease, justifying the results found here^24^.

According to the results of the miRNAs validated by RT-qPCR, only the miR-223-3p and miR-25-3p were downregulated in ACLF patients. The reason for these results may be due to the significant increase of the sample size in the validation step and by the higher sensitivity of RT-qPCR assay when compared to the microarray technique. The miR-25-3p was independently related to ACLF in the logistic regression analysis. Furthermore, miR-223-3p and miR-25-3p were negatively correlated with CLIF-SOFA score. Previous studies have shown decreased expression of circulating miR-223 in patients with hepatocellular carcinoma^25,26^, liver cirrhosis^26,27^ and chronic hepatitis C^26^. Moreover, expression of serum miR-25 has been deregulated in patients with hepatocellular carcinoma^28^. As pointed by Wasmuth *et al*.^29^, patients with severe sepsis and ACLF show a similar degree of cellular immune depression. Interestingly, low expression of miR-25-3p and miR-223-3p has already been shown in septic patients and were also related to poor prognosis^21,22^. Wang *et al*.^21^ evaluated the expression of miRNAs in 214 serum samples from sepsis patients (117 survivors and 97 non-survivors based on 28-day mortality). Six miRNAs had altered expression in patients who died, including the miR-223-3p (P < 0.001). The sensitivity and specificity of miR-223-3p to distinguish surviving patients from those who died at 28 days was similar to the SOFA score, with areas under the ROC curve (AUC) of 0.748 and 0.782, respectively. Additionally, Yao *et al*.^22^. showed that low expression of miR-25-3p was related to the severity of sepsis and 28-day mortality. Furthermore, the decreased miR-25-3p level was negatively correlated with the SOFA score, CRP, and procalcitonin level. In another study, the regulatory mechanism of miR-25-3p in sepsis-induced cardiomyocyte apoptosis was investigated. The researchers noted that the miR-25-3p expression was reduced in the serum of septic rats and in LPS-induced cardiomyocytes, while IL6 and TNF-α were increased. Furthermore, the miR-25-3p could inhibit LPS-induced cardiomyocyte apoptosis by regulating PTEN as well as reduce injury triggered by sepsis through inhibiting the inflammatory reaction^30^. The miR-233 may influence macrophage polarization, differentiation of immune cells and inflammasome activation^31^. It was suggested that increased expression of neutrophilic miR-223 in mice may protect alcohol-induced liver injury and on the other hand downregulation of neutrophilic miR-223 in alcoholics may increase alcohol-mediated activation of neutrophils and liver injury in these subjects. In addition, the decrease of miR-233 expression increased IL-6 and p47^phox^ expression in peripheral blood neutrophils from alcoholics^32^. It has been shown that miR-233 is a regulator of the progression of nonalcoholic steatohepatitis by targeting several inflammatory genes such as C-X-C motif chemokine 10 (*CXCL10*) and transcriptional co-activator with PDZ-binding motif (*Taz*) in hepatocytes^33^. The deregulation of miR-233, seen in our work, may be part of the pathogenesis of ACLF by macrophage polarization and alteration of cytokines involved in the inflammatory process.

In this study, miR-223-3p and miR-25-3p expression was negatively correlated with creatinine values and was also significantly decreased in patients with hepatic encephalopathy at admission. The miR-223-3p expression appears to be affected by renal diseases as its expression was shown to be reduced in both acute and chronic kidney diseases^34,35^. miR-25-3p was also shown to be downregulated in diabetic nephropathy, suggesting an influence of renal disease on its expression^36^. Although there are no previous studies examining the relationship between miR-223-3p, miR-25-3p, and hepatic encephalopathy, the association observed here indicate that expression of these miRNAs may be affected by the severity of the liver disease. However, kidney and cerebral dysfunction are both included in the ACLF definition. Therefore, as these miRNAs were selected from the microarray panel as ACLF markers, possibly the association with creatinine levels and hepatic encephalopathy merely reflects the severity of ACLF, as discussed above.

Expressions of miR-223-3p and miR-25-3p were associated with 30-day mortality in the univariate Cox regression analysis. However, only miR-25-3p expression remained an independent prognostic factor after adjusting for Child-Pugh classification and ACLF. In target prediction analysis it was observed that miR-25-3p might control the expression of genes related to inflammatory responses. The miR-25-3p possibily controls the translation of ICAM-1, IL6ST, IL8, TRAF3, IL15, among others. In addition, it has been observed that the predicted genes of miR-25-3p are involved with VEGF signaling pathways, TNF receptors, mTOR and TRAIL pathways. Cirrhosis-associated immune dysfunction (CAID) is an important feature of advanced liver disease and it is associated with progression to ACLF and high mortality^37^. The decrease in miR-25-3p expression in advanced cirrhosis may be responsible for an imbalance in the translation of some inflammation-related cytokines, contributing for CAID. On the other hand, a reduced miR-25-3p expression may result from systemic inflammation and, in this case, this miRNA would serve as a biomarker of immune dysfunction. However, further studies are needed to clarify the exact role of circulating miR-25-3p in CAID.

In conclusion, for the first time, ACLF was associated with a distinct miRNA profile obtained by microarray analysis of sera from patients hospitalized for acute decompensation of cirrhosis. miR-223-3p and miR-25-3p expressions were related to creatinine levels, hepatic encephalopathy, ACLF, and mortality. Circulating miR-25-3p was an independent predictor of ACLF and short-term mortality, indicating a potential as a prognostic biomarker in patients with acute decompensation of cirrhosis.

## Patients and Methods

### Study population

All patients included in the present study were admitted at the emergency room of the University Hospital of the Federal University of Santa Catarina, Brazil, from January 2013 to November 2015 due to acute decompensation of cirrhosis. This study was conducted in two phases. In the first phase, samples of 35 patients were used for miRNAs microarray analysis. In the second phase, miRNAs selected for validation by RT-qPCR in the first phase were investigated in samples of 139 patients. Patients in the following situations were excluded: (1) hospitalization for elective procedures; (2) admissions not related to complications of liver cirrhosis; (3) hepatocellular carcinoma outside Milan criteria; (4) extrahepatic malignancy; (5) severe extrahepatic disease.

The diagnosis of cirrhosis was established by histology (when available) or by the combination of imaging, biochemical and clinical findings in patients with evidence of portal hypertension.

This study was approved by the Ethics Committee on Human Research of the Federal University of Santa Catarina (approval number 948.198) and the study protocol was performed conform to the ethical guidelines of the 1975 Helsinki Declaration. We declare that the informed consent was obtained from all study participants and/or their legal guardians.

### Patients

All patients admitted for acute decompensation of cirrhosis, as defined by the acute development of hepatic encephalopathy, large ascites, gastrointestinal bleeding, bacterial infection or any combination of these, were screened. Patients were evaluated within 24 hours of admission by one of the researchers involved in the study and followed during their hospital stay. Thirty-day mortality was evaluated by phone call, in case of hospital discharge.

Active alcoholism was defined as an average overall consumption of 14 or more drinks per week for women, and 21 or more drinks per week for men during the 4 weeks before enrollment (one standard drink is equal to 12 g absolute alcohol)^38^.

Hepatic encephalopathy was graded according to the West-Haven criteria^39^. The ACLF definition was based on the EASL-CLIF Consortium definition^4^, therefore, no ACLF are patients with no organ failure or with a single “non-kidney” organ failure who had a serum creatinine level less than 1.5 mg/dL and no hepatic encephalopathy, or patients with single cerebral failure who had a serum creatinine level less than 1.5 mg/dL. ACLF are patients with single kidney failure, or patients with single failure of the coagulation, liver, circulation, respiration who had a serum creatinine level between 1.5 to 1.9 mg/dL and/or mild to moderate hepatic encephalopathy, or patients with single cerebral failure who had a serum creatinine level between 1.5 and 1.9 mg/dL or patients with two or more organ failures. Organ failure was established based on the CLIF-SOFA criteria^4^, and the severity of liver disease was estimated by the Child-Pugh^40^ and Model for End-Stage Liver Disease (MELD)^41^ classification system, calculated based on clinical and biochemical parameters obtained at admission.

### Blood collection and serum isolation

Peripheral blood was collected in clot activator and separator gel (BD Vacutainer SST II Advance) tube. The blood was centrifuged at 3000 × g for 10 min and the serum was transferred to microtubes and stored at −80 °C. Prior to use, the serum was thawed and centrifuged at 16.000 × g for 10 min at 4 °C^42^. This step allowed the removal of cells debris and cryoprecipitate. Visibly hemolyzed, icteric or lipemic samples were not used in this study.

### RNA extraction

Total RNA was extracted using the mirVana PARIS RNA kit, according to the manufacturer instructions (Life Technologies, California, USA) using 500 μL of serum. After the denaturation, 25 fmol of synthetic miRNA cel-miR-238^43^ (where “cel” corresponds to the abbreviation of species *Caenorhabditis elegans*) was added, which was used as quality control of RNA extraction and RT-qPCR.

### miRNA microarray

miRNA expression profiling was performed using Human Agilent’s miRNA Microarray system (Agilent Technologies, California, USA) with probe sets for human miRNAs (miRBase release 21). In brief, 35 μL of total RNA was concentrated in SpeedVac™ Concentrator Savant SPD1010 (Thermo Fisher Scientific, Massachusetts, USA) at room temperature and vacuum pressure of approximately 10 Torr. The total RNA was fluorescence-labeled with Cyanine 3 and hybridized onto the arrays for 20 h at 55 °C. Slides were scanned in a SureScan Microarray Scanner (Agilent Technologies, California, USA) and the images obtained were processed with Feature Extraction Software (Agilent Technologies, California, USA). Intensity values were processed using Genespring software (Agilent Technologies, California, USA) for evaluation of the altered miRNAs. The microarray data were shown as fold change (FC) value, which describes the ratio of miRNA expression between the groups studied.

### Nomenclature of miRNAs

The denomination of the miRNAs was done according to the miRBase database (www.mirbase.org) with the exception of the term “hsa” (abbreviation of species *Homo sapiens*) in front of the names, to avoid repetitions, as all samples are from human source.

### Reverse transcription-quantitative polymerase chain reaction (RT-qPCR)

Expression of selected mature miRNAs was assessed by RT-qPCR. First, 35 μL of total RNA was concentrated in SpeedVac™ Concentrator Savant SPD1010 (Thermo Fisher Scientific, Massachusetts, USA) for 1 h at room temperature and vacuum pressure of approximately 10 Torr. The polyadenylation and cDNA were prepared from total RNA using miRNA RT-qPCR Master Mix Detection Kit (Agilent Technologies, California, USA) in a Veriti Thermal Cycler (Applied Biosystems, California, USA). The qPCR was done using a StepOnePlus Real-Time PCR System (Applied Biosystems, California, USA) to quantify the levels of cel-miR-238-3p; miR-223-3p; miR-20a-5p; miR-106b-5p; miR-25-3p; miR-126-3p; and miR-1273g-3p. All reactions were run in duplicate and normalized to the expression of miR-1273g-3p. The relative expression of the miRNAs was calculated by the comparative quantification cycle (Cq) method^44^, where the Cq is the PCR cycle number at which the sample fluorescence signal passes a fixed threshold line, and reported as 2^−ΔCq (miR-target – miR-1273g-3p)^.

### Bioinformatic analysis

The bioinformatic analysis was performed using the starBase software version 2.0^45^. This program compiles the miRNA-mRNA interaction data from five prediction programs: TargetScan (www.targetscan.org), PicTar (pictar.mdc-berlin.de), PITA (https://genie.weizmann.ac.il/pubs/mir07/mir07_prediction.html), miRanda (www.microrna.org) and RNA22 (https://cm.jefferson.edu/rna22/Precomputed/). Furthermore, the enrichment analysis of the biological pathways was performed using the FunRich software version 3.1.3^46^.

### Statistical analysis

Data from miRNA microarray were analyzed by Genespring GX (Agilent Technologies, California, USA) and GraphPad Prism 6 (GraphPad Software, California, USA) software. Normality of the variable distribution was determined using the Kolmogorov-Smirnov test. The miRNAs with Fold Change (FC) ≥ 6 were identified by the non-parametric Mann-Whitney U test with Benjamini-Hochberg correction to compare no ACLF with ACLF patients, and Storey with bootstrapping correction was used to compare patients according to the occurrence or not of death in 30 days.

Data produced from the RT-qPCR were analyzed in the IBM SPSS Statistics version 22 software (IBM, Chicago, IL, USA). The Normality of the variable distribution was also evaluated by the Kolmogorov-Smirnov test. Continuous variables were compared using Student’s t-test in the case of a normal distribution or Mann-Whitney test in the remaining cases. The correlation between the numerical variables was evaluated using Spearman’s correlation coefficient (ρ Spearman). Categorical variables were assessed using the chi-square test. Multiple logistic regression analysis (forward conditional method) was used to investigate factors independently associated with ACLF. Univariate and multivariate Cox regression analyses were used to investigate the association of miRNA expression with 30-day mortality. The best cutoff points for the miRNAs to predict mortality were chosen by the Receiver Operating Characteristics (ROC) curve. The Kaplan-Meier curve was used to demonstrate 30-day survival according to two strata, defined by the cutoff of selected miRNAs. Survival differences between groups were compared using the log-rank test. P-values less than 0.05 were considered statistically significant.

## Supplementary information

Supplementary information.

## Data Availability

The datasets generated and analyzed during the current study are contained within the manuscript and in supplementary material. Additional raw data can be available upon request.
