# Supplementary material for: MicroRNA profiles in serum samples from Acute-On-Chronic Liver Failure patients and miR-25-3p as a potential biomarker for survival prediction
Source: Sci Rep. 2020 Jan 9;10:100. doi: 10.1038/s41598-019-56630-5 (PMC6952390; doi:10.1038/s41598-019-56630-5)
Supplement: Supplementary file 1 — Supplementary information. [file 41598_2019_56630_MOESM1_ESM.pdf]

SUPPLEMENTARY INFORMATION

MicroRNA profiles in serum samples from Acute-On-Chronic Liver Failure patients and miR-25-3p as a potential biomarker for survival prediction

Júlia Cisilotto[1]; Alex Evangelista do Amaral[1]; Daiane Rosolen[1]; Michele Patrícia Rode[1]; Adny Henrique Silva[2]; Evelyn Winter[3]; Telma Erotides da Silva[4]; Josiane Fischer[4]; Camila Matiollo[4]; Elayne Cristina de Morais Rateke[4]; Janaína Luz Narciso-Schiavon[4], Leonardo de Lucca Schiavon[4]; Tânia Beatriz Creczynski-Pasa[1].

[1]Department of Pharmaceutical Sciences, Federal University of Santa Catarina, Florianopolis, 88040-900, SC, Brazil

[2]Department of Biochemistry, Federal University of Santa Catarina, Florianopolis, 88040-900, SC, Brazil

[3]Department of Agriculture, Biodiversity and Forestry, Federal University of Santa Catarina, Curitibanos, 89520-000, SC, Brazil

[4]Department of Internal Medicine, Division of Gastroenterology, Federal University of Santa Catarina, Florianópolis, 88040-900, SC, Brazil

***Corresponding Authors:**

Tânia Beatriz Creczynski-Pasa

Tel/Fax: (+55 (48) 32212200

e-mail: tania.pasa@ufsc.br

Leonardo de Lucca Schiavon

Tel/Fax: (+55 (48) 32096854

e-mail: leo-jf@uol.com.br

Figure S1 are shown the miRNAs expressed on patients who used in relation who not used medications treatment at admission in the hospital. Table S1 are shown the miRNAs expression and their association with different variables in liver disease and Table S2 the Spearman's correlation coefficient between miRNAs and biochemical parameters associated with liver disease. Furthermore, in Figure S2 it is observed the biological pathway enrichment analysis of miR-25-3p target genes.

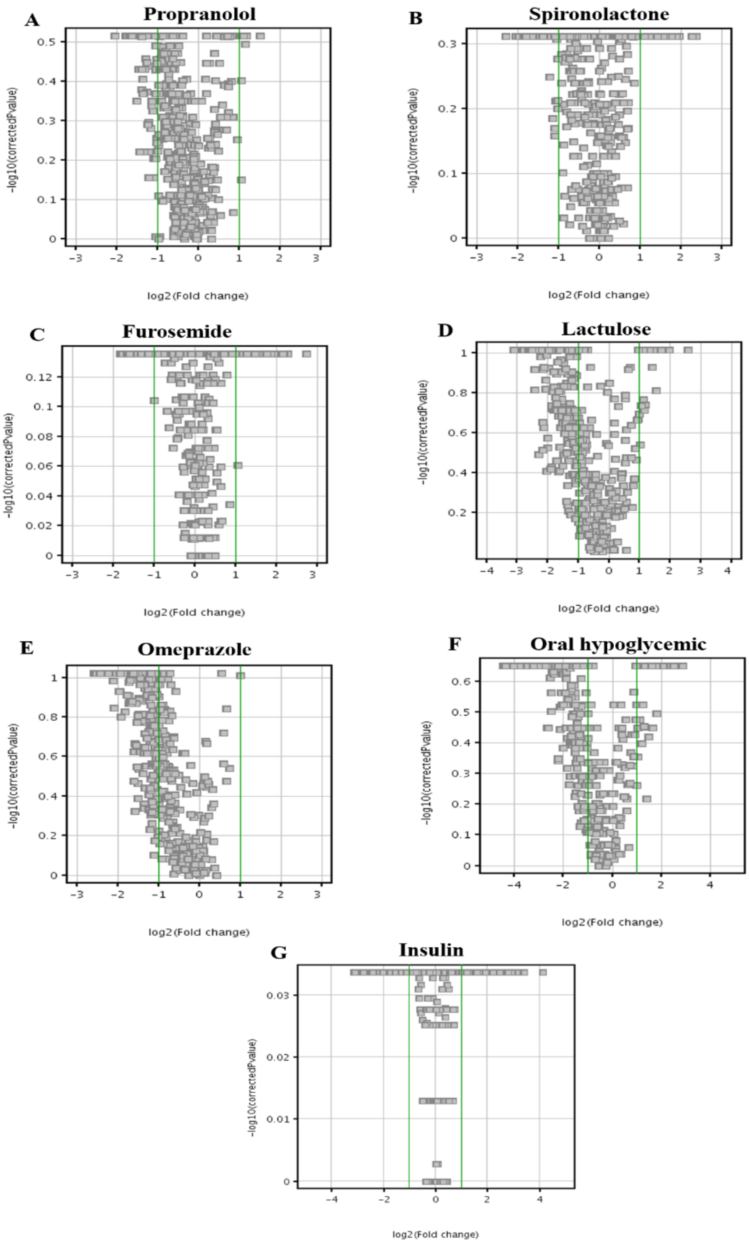

Figure S1. Volcano plot of miRNAs expressed after a Mann-Whitney U test and Benjamin Hochberg post-hoc test (fold change > 2 and P-values < 0.05) on patients who used or not propranolol (A); spironolactone (B); furosemide (C); lactulose (D); omeprazole (E), oral hypoglycemic (F), insulin (G).

Table S1. miRNAs expression and their association with different studied variables.

Parameters	P-value miRNA				
	**106b-5p**	**126-3p**	**20a-5p**	**223-3p**	**25-3p**
**Gender**	0.595	0.963	0.923	0.671	0.339
**Etiology of cirrhosis**					
Hepatitis B	0.218	0.171	0.782	0.328	0.320
Hepatitis C	0.910	0.934	0.931	0.566	0.879
Alcohol	0.374	0.354	0.781	0.940	0.402
**Medications use at admission**					
Propranolol	0.763	0.974	0.979	0.409	0.538
Spironolactone	0.532	0.830	0.657	0.439	0.762
Furosemide	0.081	0.132	0.161	0.802	0.346
Lactulose	0.413	0.237	0.641	0.988	0.597
Norfloxacin	0.518	0.812	0.869	0.649	0.864
Omeprazole	0.132	0.317	0.269	0.206	0.422
Oral hypoglycemic*	0.330	0.425	0.943	0.885	0.580
Insulin	0.455	0.821	0.380	0.242	0.664
**Child-Pugh C**	0.615	0.338	0.907	0.150	0.338
**Bacterial infection (first 48 h)**	0.500	0.587	0.522	0.587	0.794
**Gastrointestinal bleeding (first 48 h)**	0.199	0.082	0.199	0.202	0.125
**Complication at admission**					
Ascites	0.765	0.833	0.899	0.484	0.333
Hepatic encephalopathy	0.402	0.203	0.203	0.005**	0.034*
**Organ failures**					
Kidney	0.987	0.885	0.403	0.218	0.131
Hepatic	0.342	0.467	0.187	0.130	0.057
Brain	0.235	0.127	0.271	0.020*	0.073
Coagulation	0.706	0.873	0.919	0.805	0.717
Circulatory	0.550	0.165	0.596	0.636	0.995
**ACLF**	0.597	0.362	0.258	0.026*	0.009**

ACLF. acute-on-chronic liver failure; *metformin, glibenclamide and mlimepiride. Statistical differences were calculated using Mann-Whitney U test. *P<0.05, **P<0.01.

Table S2. Spearman's correlation coefficient between miRNAs and biochemical parameters associated with liver disease.

**Variables**		**miRNA**				
		**106b-5p**	**126-3p**	**20a-5p**	**223-3p**	**25-3p**
Age	r	-0.165	-0.133	-0.127	-0.193*	-0.106
	P-value	0.056	0.125	0.143	0.025	0.220
Creatinine	r	-0.140	-0.096	-0.212*	-0.302***	-0.341***
	P-value	0.101	0.259	0.012	<0.001	<0.001
Total leukocyte	r	-0.212*	-0.074	-0.166	-0.021	-0.153
	P-value	0.012	0.384	0.051	0.803	0.072
ALP	r	-0.010	0.017	0.098	0.062	0.019
	P-value	0.902	0.843	0.249	0.471	0.823
GGT	r	-0.126	0.026	-0.043	0.055	-0.028
	P-value	0.141	0.760	0.615	0.519	0.745
Total bilirubin	r	-0.051	-0.028	-0.046	-0.065	-0.111
	P-value	0.556	0.744	0.591	0.452	0.198
Albumin	r	0.057	0.027	0.054	0.054	0.025
	P-value	0.515	0.757	0.538	0.535	0.773
INR	r	0.056	0.072	0.050	0.060	0.030
	P-value	0.518	0.406	0.565	0.488	0.731
Sodium	r	-0.041	-0.101	-0.050	-0.006	0.016
	P-value	0.640	0.245	0.563	0.946	0.857
CRP	r	-0.080	-0.038	-0.059	0.012	-0.056
	P-value	0.373	0.669	0.511	0.891	0.529
Mean blood pressure	r	-0.001	-0.086	-0.014	0.058	0.092
	P-value	0.994	0.325	0.871	0.508	0.292
CLIF-SOFA	r	-0.079	-0.092	-0.107	-0.186*	-0.196*
	P-value	0.370	0.296	0.224	0.034	0.026
Child-Pugh pontuation	r	-0.050	-0.088	-0.065	-0.130	-0.130
	P-value	0.559	0.308	0.448	0.129	0.131
MELD score	r	-0.070	-0.049	-0.089	-0.150	-0.185*
	P-value	0.414	0.567	0.296	0.078	0.029

ALP. alkaline phosphatase; GGT. γ-glutamyl transpeptidase; INR. international normalized ratio; CRP. C-reactive protein; MELD. Model for End-stage Liver Disease; r. Spearman's correlation coefficient; *P<0.05; ***P<0.001.

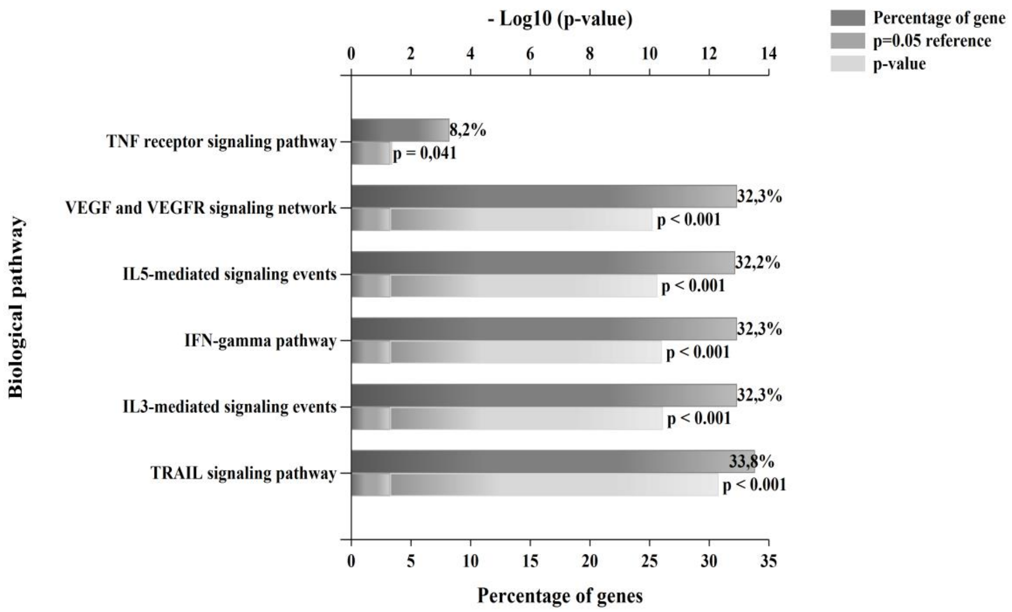

Figure S2. Biological pathway enrichment analysis of miR-25-3p target genes.

TNF. tumor necrosis factor; VEGF. vascular endothelial growth factor; VEGFR. vascular endothelial growth factor receptor; IL5. interleukin 5; INF. interferon; IL3. interleukin 3; TRAIL. tumor necrosis factor-related apoptosis-inducing ligand.
